# ABA‐mediated regulation of stomatal density is OST1‐independent

**DOI:** 10.1002/pld3.82

**Published:** 2018-09-19

**Authors:** Pirko Jalakas, Ebe Merilo, Hannes Kollist, Mikael Brosché

**Affiliations:** ^1^ Institute of Technology University of Tartu Tartu Estonia; ^2^ Viikki Plant Science Centre, Organismal and Evolutionary Biology Research Programme Faculty of Biological and Environmental Sciences University of Helsinki Helsinki Finland

**Keywords:** abscisic acid, aperture width, OST1, signaling pathways, Stomatal density

## Abstract

Stomata, small pores on the surfaces of leaves formed by a pair of guard cells, adapt rapidly to changes in the environment by adjusting the aperture width. As a long‐term response, the number of stomata is regulated during stomatal development. The hormone abscisic acid (ABA) regulates both processes. In ABA mediated guard cell signaling the protein kinase OPEN STOMATA1 (OST1) has a central role, as stomatal closure in the *ost1* mutant is impaired in response to ABA and to different environmental stimuli. We aimed to dissect the contribution of different ABA‐related regulatory mechanisms in determining stomatal conductance, a combination of stomatal density and aperture width, and crossed the *ost1* mutant with mutants that either decreased (*aba3*) or increased (*cyp707a1/a3*) the concentration of ABA in plants. The double mutant *ost1 aba3* had higher stomatal conductance than either parent due to a combination of increased stomatal aperture width and higher stomatal density. In the triple mutant *ost1 cyp707a1/a3*, stomatal conductance was significantly lower compared to *ost1‐3* due to lower stomatal density. Further characterization of the single, double and triple mutants showed that responses to treatments that lead to stomatal closure were impaired in *ost1* as well as *ost1 aba3* and *ost1 cyp707a1/a3* mutants, supporting a critical role for OST1 in stomatal aperture regulation. On the basis of our results, we suggest that two signaling pathways regulate water flux from leaves, that is, stomatal conductance: an ABA‐dependent pathway that determines stomatal density independent of OST1; and an OST1‐dependent pathway that regulates rapid changes in stomatal aperture.

## 1 INTRODUCTION

Stomata, formed by a pair of guard cells, are small pores responsible for gas exchange in leaves. They allow CO_2_ uptake for photosynthesis, with the accompanying loss of water. In addition, air pollutants and some pathogens enter the plant through stomata. Hence, accurate adjustment of the stomatal pore is required for the plant to successfully thrive in a changing environment. Not only the width of the stomatal aperture, but also the number of stomata is regulated by environmental signals and influences plant gas exchange (Hetherington & Woodward, 2003). Several studies have shown that doubling of ambient CO_2_ concentration leads to a reduction in stomatal density in different plant species and *Arabidopsis* accessions (Woodward & Kelly, 1995; Woodward, Lake, & Quick, 2002). In contrast, higher light intensity significantly increases stomatal density (Casson et al., 2009). The main difference between the adjustments of the stomatal aperture versus the number of stomata is the scale of time, stomatal aperture can change in minutes, whereas changes in stomatal density are fixed during leaf development.

The plant hormone abscisic acid (ABA) plays a central role in the regulation of guard cell function (Kim, Böhmer, Hu, Nishimura, & Schroeder, 2010; Kollist, Nuhkat, & Roelfsema, 2014). ABA‐induced stomatal closure is initiated by binding of the hormone to PYR/RCAR receptors that leads to the inactivation of type 2C protein phosphatases (PP2Cs), which in turn releases SNF‐related protein kinases (SnRK2s) such as OPEN STOMATA1 (OST1) to activate guard cell ion channels including SLOW ANION CHANNEL1 (SLAC1). This leads to the efflux of anions, followed by potassium and water efflux and stomatal closure (Kim et al., 2010; Kollist et al., 2014).

As ABA is the central regulatory molecule of stomatal function, fine‐tuning of ABA levels and signaling is of utmost importance during acclimation to abiotic stress, for example, drought. Guard cell ABA levels are regulated by de novo biosynthesis, catabolism, and transport from other plant tissues (Merilo et al., 2015, 2018; Nambara & Marion‐Poll, 2005). OST1 appears to have a critical role in ABA signaling. Stomatal closure induced by ABA or environmental factors is strongly impaired in *ost1* mutants (Merilo et al., 2013; Mustilli, Merlot, Vavasseur, Fenzi, & Giraudat, 2002). In addition to stomatal regulation, ABA affects stomatal development, which is also controlled by environmental factors such as light and the level of CO_2_ (Casson & Hetherington, 2010; Chater et al., 2015). ABA‐deficient mutants have increased stomatal densities compared to wild‐type (Chater et al., 2015; Tanaka, Nose, Jikumaru, & Kamiya, 2013), whereas the ABA over‐accumulating *cyp707a1/a3* double mutant had significantly lower stomatal density than wild‐type (Tanaka et al., 2013), supporting the role of ABA in stomatal development. ABA3 encodes a molybdenum cofactor sulfurase required by an abscisic aldehyde oxidase to catalyze the conversion of abscisic aldehyde to ABA; its expression level increases in response to drought and ABA treatment (Xiong, Ishitani, Lee, & Zhu, 2001). In non‐stressed conditions, the concentration of leaf ABA is approximately 45% of wild‐type ABA in *aba3‐1* (Merilo et al., 2018). The predominant ABA catabolic pathway, ABA 8′‐hydroxylation, is mediated by four members of the CYP707A gene family and their transcription levels increase in response to salt and drought stress as well as ABA (Saito et al., 2004). CYP707A1 and CYP707A3 are important for post‐germination growth, since seedling growth by exogenous ABA was inhibited more effectively in *cyp707a1* and *cyp707a3* mutants and was more pronounced in the double mutant that also contained higher concentration of ABA compared to the single mutants (Okamoto et al., 2006). Both *cyp707a1* and *cyp707a3* loss‐of‐function mutants showed reduced stomatal conductance, which was more pronounced in *cyp707a3* (Merilo et al., 2013).

Here, we report that while OST1 is required in rapid stomatal responses to several environmental conditions: reduced air humidity, darkness, elevated CO_2_ concentration and exogenous ABA, OST1 is not required for regulation of stomatal density. These two pathways, OST1‐dependent signaling that regulates stomatal aperture width and OST1–independent signaling that regulates stomatal density, coordinate the overall water flux through stomata.

## 2 MATERIALS AND METHODS

### 2.1 Plant material, growth and gas‐exchange measurements

Col‐0, *aba3‐1* and *ost1‐3* (*srk2e*, SALK_008068) were from the European Arabidopsis Stock Centre (www.arabidopsis.info). The *cyp707a1 cyp707a3* double mutant was a gift from Eiji Nambara (Okamoto et al., 2006). Double mutants and other crosses were made through standard techniques and genotyped with PCR‐based markers.

**Table:** 

*aba3‐1*	aba3‐1‐left	TCATTCTTTCTACTGCTCCTGATTT	dCAPS marker, digest with MnlI
	aba3‐1‐right	GGTGAAGCAAATGAACTTATGATG	
*ost1‐3*	SALK_008068 for	CCTCTGATGTCTTGGTGTCG	
	SALK_008068 rev	TGGAAGAAAAACCTCGCCTA	
*cyp707a1*	SALK_069127 for	CATGAACGTATTGGGTTTTGG	
	SALK_069127 rev	TCCTGATATTGAATCCATCGC	
*cyp707a3*	SALK_078173C for	GTTCCTGGAAGATTAATCGGC	
	SALK_078173C rev	ACGTGCTCTCGTCACTCTCTC	
SALK Lbb		GCGTGGACCGCTTGCTGCAACT	

Plants for gas‐exchange measurements were sown into 2:1 (v:v) peat:vermiculite mixture and grown through a hole in a glass plate covering the pot as described in Kollist et al. (2007). Plants were grown in growth chambers (AR‐66LX, Percival Scientific, IA, USA and Snijders Scientific, Drogenbos, Belgia) with 12 hr photoperiod, 23/18°C day/night temperature, 150 μmol m^−2^ s^−1^ light and 70% relative humidity. Plants were 24–30 days old during gas‐exchange experiments.

Stomatal conductance of intact plants was measured using a rapid‐response gas‐exchange measurement device similar to the one described by Kollist et al. (2007) consisting of eight thermostated flow‐through whole‐rosette cuvettes. Plants were inserted into the measurement chambers and after stomatal conductance had stabilized, the following stimuli were applied: reduction in air humidity (decrease from 65%–75% to 30%–40%), darkness (decrease from 150 μmol m^−2^ s^−1^ to 0 light), CO_2_ (increase from 400 ppm to 800 ppm) and spraying plants with 0 μM, 5 μM or 50 μM ABA solution with 0.012% Silwet L‐77 (Duchefa) and 0.05% ethanol. ABA‐induced stomatal closure experiments were carried out as described previously (Merilo et al., 2018). Initial changes in stomatal conductance were calculated as gs18‐gs0, where gs0 is the pretreatment stomatal conductance and gs18 is the value of stomatal conductance 18 min after factor application; 16 min in case of ABA spraying.

### 2.2 Measurement of stomatal aperture and density

Epidermal peels were stripped from 4‐week‐old plants grown in growth chambers as described above and incubated in resting buffer (containing 10 mM MES‐KOH pH6.2) for 2.5 hr. Images of stomata were taken with a Zeiss Axio Examiner.D1 microscope. Images were taken of 15 stomata per leaf and averaged to characterize the stomatal aperture of each plant. Six plants per genotype were analyzed. Stomatal aperture width was measured using the image processing software ImageJ 1.51k (National Institutes of Health, USA).

For stomatal density (SD) measurements, leaves of 5 week‐old plants grown as described above, one leaf per plant, were excised and the abaxial side was covered with dental resin (Xantropen VL Plus, Heraeus Kulzer, Germany). The hardened resin impressions were covered with transparent nail varnish. The dried nail varnish imprints were attached to a microscope glass slide with a transparent tape and images were taken with a Zeiss SteREO Discovery.V20 stereomicroscope. Twenty‐four plants per genotype were analyzed. SD was determined from an image with an area of ~0.12 mm^2^, taken from the middle of the leaf, close to the middle vein and calculated as: *SD* = number of stomata/area of the image.

### 2.3 Statistical analysis

One‐way ANOVA was used to compare the effect of genotype on the values of stomatal conductance, aperture, density and initial change in stomatal conductance. Comparisons between individual means were done with Tukey or Tukey unequal N HSD *post hoc* tests as indicated in figure legends. Stomatal conductance values before and after application of ABA were compared by repeated measures ANOVA with Tukey *post hoc* test. All effects were considered significant at *p* < 0.05. Statistical analyses were performed with Statistica, version 7.1 (StatSoft Inc., Tulsa, OK, USA).

### 2.4 Accession numbers

ABA3—AT1G16540; OST1—AT4G33950; CYP707A1—AT4G19230; CYP707A3—AT5G45340.

## 3 RESULTS

**Figure 1:**
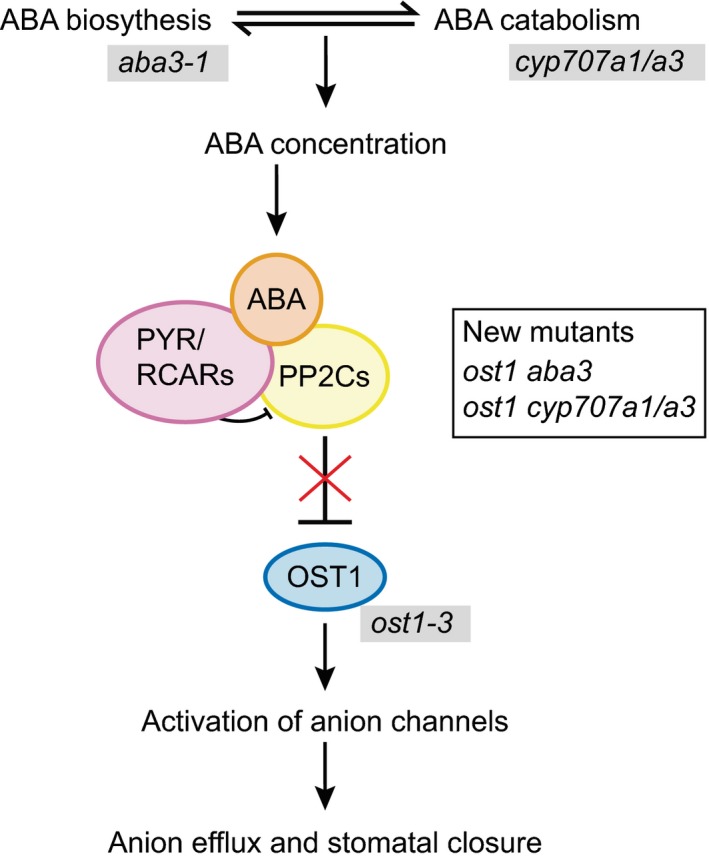
Schematic overview of ABA concentration determined by ABA biosynthesis and catabolism, followed by the core components in ABA signaling leading to stomatal closure. Mutants used in this study are indicated in gray background. New double and triple mutants generated for this study are *ost1 aba3* and *ost1 cyp707a1/a3*

**Figure 2:**
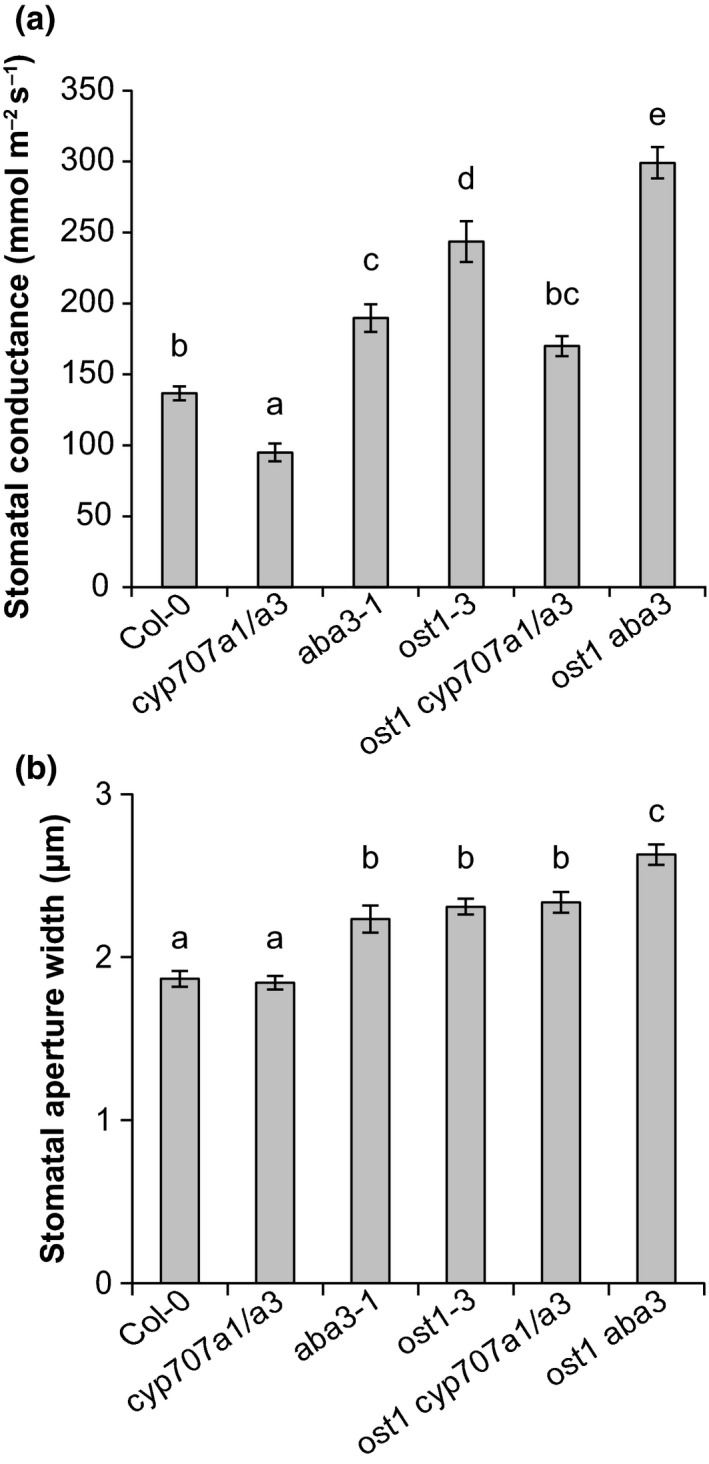
(a) Whole‐plant steady‐state stomatal conductance (gs) of 3‐ to 4‐week‐old plants. The ABA biosynthesis mutant *aba3‐1* and catabolism double mutant *cyp707a1/a3* were crossed to *ost1‐3* to genetically reduce or increase the ABA concentration in *ost1‐3* background. Letters denote statistically significant differences between lines (ANOVA with Tukey unequal N HSD *post hoc* test, *p* < 0.05; *n *= 8–13). (b) Stomatal aperture measured on epidermal peels of 4‐week‐old plants. Letters denote statistically significant differences between lines (ANOVA with Tukey *post hoc* test, *p* < 0.05; *n* = 6)

We crossed *ost1‐3* into an ABA biosynthesis mutant (*aba3‐1*) and to *cyp707a1 cyp707a3* (here abbreviated as *cyp707a1/a3*) that lacks two proteins involved in ABA catabolism. By doing so, we generated plants where strong ABA‐insensitivity caused by impaired OST1 was combined with defective ABA biosynthesis or breakdown (Figure 1). Steady‐state stomatal conductance and rapid stomatal responses to various closure‐inducing stimuli were measured in intact plants with a custom‐made gas‐exchange device as described before (Kollist et al., 2007). Our results showed that the *aba3‐1* mutant displayed higher stomatal conductance, whereas *cyp707a1/a3* had reduced stomatal conductance compared to Col‐0 wild‐type (Figure 2a), as can be expected on the basis of the ABA concentrations in these plants (Merilo et al., 2018; Okamoto et al., 2006). The double mutant *ost1 aba3* had higher stomatal conductance than either parent (Figure 2a) and the triple mutant *ost1 cyp707a1/a3* displayed lower stomatal conductance than the single *ost1‐3* (Figure 2a).

As altered stomatal conductance can result from a change in stomatal aperture width or in stomatal density, we next determined the stomatal apertures of the mutants. There were no differences in aperture widths between *cyp707a1/a3* and wild‐type (Figure 2b). Compared to wild‐type, stomata of *aba3‐1* and *ost1‐3* single mutants had significantly wider apertures (Figure 2b). Aperture of *ost1 cyp707a1/a3* was similar to *ost1‐3*, whereas *ost1 aba3* had significantly wider aperture compared to the single mutants (Figure 2b). These results suggest that ABA‐deficiency leads to wider stomatal apertures, whereas over‐accumulation of ABA seems to have no effect on aperture width. However, *cyp707a1/a3* (compared to Col‐0) and *ost1 cyp707a1/a3* (compared to *ost1*) showed differences in stomatal conductance but not in aperture widths, indicating that some other trait besides aperture is involved in determining stomatal conductance.

**Figure 3:**
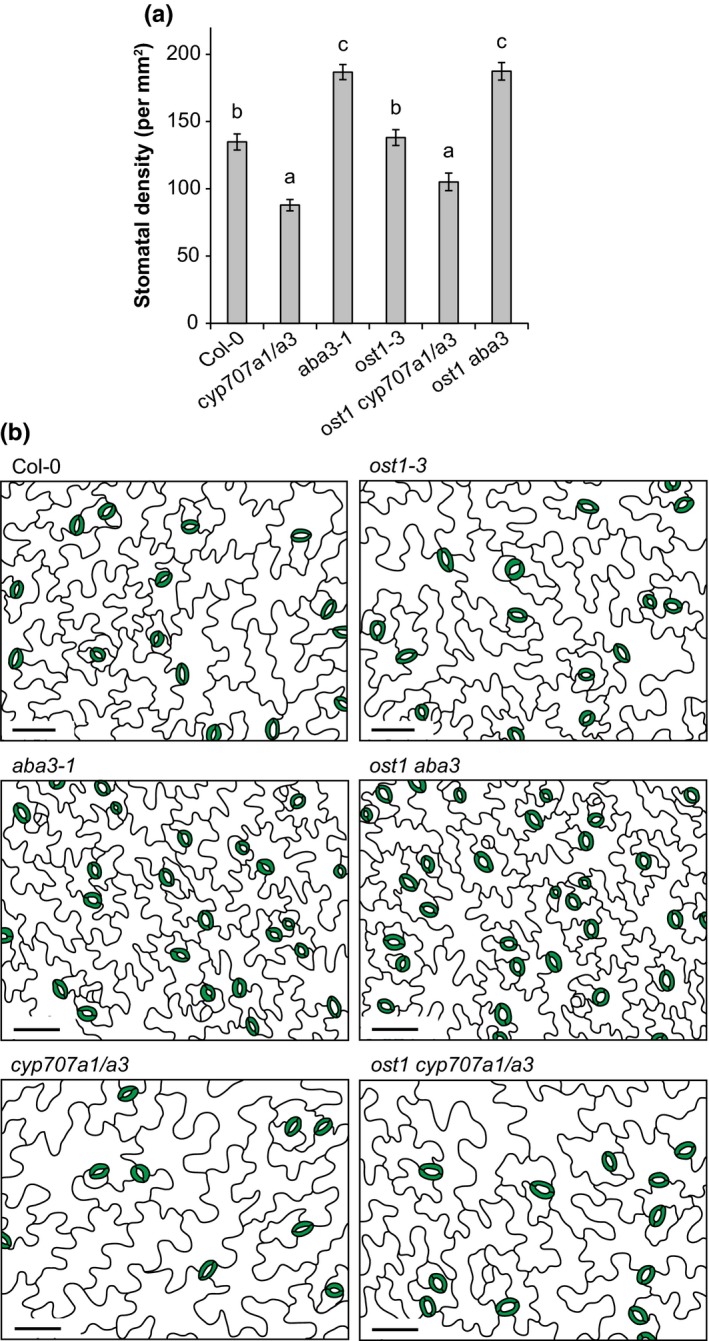
(a) Stomatal density of 5‐week‐old plants. Letters denote statistically significant differences between lines (ANOVA with Tukey *post hoc* test, *p* < 0.05; *n* = 24). (b) Tracing of epidermal impressions to illustrate the differences in stomatal densities between lines. The scale bar represents 50 μm

To test whether the differences in stomatal conductance in the studied mutants were associated with altered stomatal density, we measured stomatal density using leaf impressions. Consistent with already published results (Chater et al., 2015; Tanaka et al., 2013), *aba3‐1* had higher and *cyp707a1/a3* lower stomatal density compared to wild‐type in our experiment (Figure 3a,b). The stomatal density of *ost1‐3* was similar to wild‐type, but through genetically altering the ABA concentration in the *ost1‐3* mutant, we could affect the stomatal development. Compared to the single *ost1‐3* mutant, *ost1 aba3* and *ost1 cyp707a1/a3* had significantly higher or lower stomatal density, respectively (Figure 3a,b). Taken together, stomatal conductance, aperture and density results show that *ost1‐3* has higher stomatal conductance due to more open stomata (Figures 2, 3; Mustilli et al., 2002). However, ABA concentration is a crucial signal for stomatal development, which was apparently regulated by an OST1‐independent mechanism.

**Figure 4:**
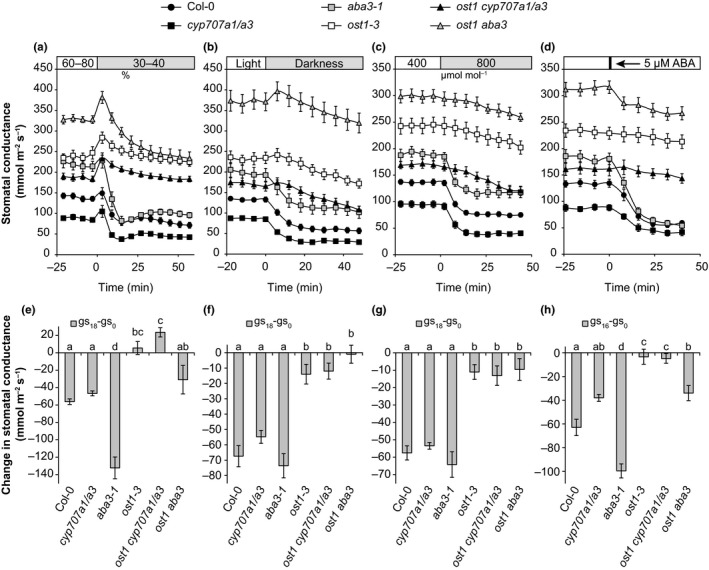
(a–d) Time courses of stomatal conductances in response to reduced air humidity (a), darkness (b), elevated CO_2_ (c) and ABA treatment (d). (e–h) Changes in stomatal conductance during the first 18 min (first 16 min in the ABA treatment). Letters denote statistically significant differences between lines (ANOVA with Tukey unequal N HSD *post hoc* test, *p* < 0.05; *n* = 8–15)

To further characterize the role of ABA levels and OST1 in stomatal regulation, we tested the responses of single mutants, *ost1 aba3* and *ost1 cyp707a1/a3* to closure‐inducing factors (Figure 4a–d). The closure induced by all stimuli was significantly impaired in *ost1‐3*, whereas *aba3‐1* plants showed wild‐type‐like closure or, in the case of reduced air humidity and ABA, even a hypersensitive response. In response to darkness, reduced air humidity and elevated CO_2_, the behavior of *ost1 aba3* and *ost1 cyp707a1/a3* mutants was not significantly different from *ost1‐3* single mutant (Figure 4a–d, e–h). In response to ABA, *ost1 aba3* plants regained a small response that was larger compared to *ost1‐3*, but reduced compared to wild‐type (Figure 4d,h). Nevertheless, *ost1 aba3* and *ost1 cyp707a1/a3* were clearly impaired in rapid stomatal responses, supporting the critical role of OST1 in the regulation of stomatal aperture to sudden changes in the environment.

## 4 DISCUSSION

Understanding stomatal function is critical for breeding plants with improved properties in water limiting conditions. As increased water loss from plants could be the result of either more open stomata or an increased number of stomata, the regulatory interplay between these traits is an important issue to be resolved. Changes in stomatal aperture width and density resulted in altered stomatal conductance, as suggested by our results (Figures 2, 3a). Through genetic manipulation of ABA levels, measurements of stomatal conductance, aperture, density, and responses to various treatments, we propose that the overall water flux through stomata is the sum of two signaling pathways: OST1‐independent regulation of stomatal density; and an OST1‐dependent pathway that regulates rapid changes of stomatal aperture. This conclusion is supported by our results which show significant differences in stomatal conductance due to differences in stomatal density and aperture between the single *ost1‐3* mutant and its double and triple mutants, where ABA levels are genetically reduced (*ost1 aba3*) and increased (*ost1 cyp707a1/a3*), respectively, while responses to various environmental stimuli are impaired in these mutants. It remains to be clarified whether other SnRKs besides OST1 (SnRK2.6) are involved in the ABA‐dependent signaling involved in stomatal development. Alternatively, stomatal density could be determined by a SnRK‐independent mechanism. However, the recent findings that a mutant lacking six ABA receptors and the PP2C mutants *abi1‐1* and *abi2‐1* also showed higher stomatal densities (Merilo et al., 2018; Tanaka et al., 2013) indicate that the canonical ABA signaling pathway starting with ABA receptors (Figure 1) is involved in the regulation of stomatal development.

The change in stomatal conductance of mutants with altered concentrations of ABA appears to result from a change in stomatal density (Figure 3a), aperture width or both, as in *aba3‐1* (Figure 2). The *aba3‐1* mutant is relatively mildly impaired in ABA biosynthesis and still contains approximately 45% of wild‐type ABA levels (Merilo et al., 2018). Mutants with more severely impaired ABA biosynthesis including *aba2‐11* or *nced3 nced5* have considerably higher stomatal conductance than *aba3‐1* (Merilo et al., 2018). Thus, the influence of ABA on stomatal aperture or density might become more prominent in plant lines where ABA concentrations are more severely reduced. Using stronger ABA‐deficient lines including *aba2‐11* or *nced3 nced5* (Merilo et al., 2018) or growing plants in water deficit conditions and measuring aperture and density may help to understand the balance between stomatal density and aperture in determining water flux through plants, that is, stomatal conductance. Stomatal density appeared to be more sensitive to reduced ABA levels than to increased levels as *cyp707a1/a3* showed only reduced density compared to wild‐type (Figure 2b). To breed crops for future climate, we need to understand the contribution of both stomatal density and stomatal aperture to plant water relations (see also Hughes et al., 2017). The latter is subjected to a rapid and up‐to‐date environmental control, whereas the former is fixed during plant development.

The triple mutant *snrk2.2 snrk2.3 snrk2.6* is completely impaired in ABA responses, including seed germination and gene expression (Fujii & Zhu, 2009; Umezawa et al., 2009). Thus, the OST1‐independent mechanism regulating stomatal density could be genetically redundant among this group of SnRKs. Unfortunately the severe developmental defects of the *snrk* triple mutant (Fujii & Zhu, 2009) make it difficult to directly test this hypothesis. The *ost1* mutant was previously shown to completely lack stomatal responses to ABA (Mustilli et al., 2002), and applied at 5 μM, the *ost1* mutant is unresponsive to ABA (Figure 4d). In an attempt to clarify if there is genetic redundancy among the SnRKs also in stomatal function, we treated *ost1‐3* plants with very high 50 μM ABA, which induced a partial stomatal closure (Figure S1). This supports earlier findings that besides OST1 there are other components, including SNRK2.2 and SNRK2.3 and other possible kinases, such as calcium dependent protein kinases (Brandt et al., 2015) or GHR1 (GUARD CELL HYDROGEN PEROXIDE RESISTANT1) (Hua et al., 2012), that might contribute to ABA‐induced stomatal closure. Other kinases may also explain the increased aperture width of *ost1 aba3* double mutant compared to single mutants. Several mitogen‐activated protein kinases (MAPK) are involved in stomatal development (Wang, Ngwenyama, Liu, Walker, & Zhang, 2007) and ABA signaling (Jammes et al., 2009), indicating that MPKs might also contribute in the regulation of stomatal development and aperture in an ABA‐dependent manner. Therefore, there are several candidate kinases in addition to OST1 in regulating stomatal responses to ABA.

Our results presented here show that it is possible to separate the regulation of stomatal aperture versus stomatal development. This information can be useful to breed separately for these traits to obtain plants suited for either rapidly changing environmental conditions or for conditions characterized by long‐term drought.

## CONFLICT OF INTEREST

No conflict of interest declared.

## AUTHOR CONTRIBUTIONS

M.B. conceived the project with help from H.K. and E.M., P.J. performed most of the experiments with assistance from E.M. and M.B., E.M. and M.B. supervised the experiments. P.J., E.M., H.K., and M.B. analyzed the data. P.J. and M.B. wrote the article with input from all authors.
