# Supplementary material for: ABA‐mediated regulation of stomatal density is OST1‐independent
Source: Plant Direct. 2018 Sep 19;2(9):e00082. doi: 10.1002/pld3.82 (PMC6508810; doi:10.1002/pld3.82)
Supplement: Supplementary file 1 [file PLD3-2-e00082-s001.pdf]

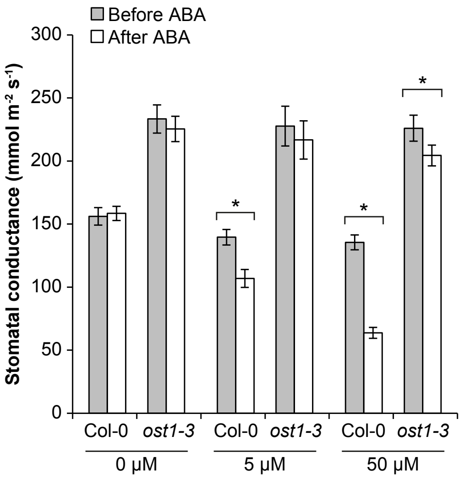

Figure S1. Stomatal response of Col-0 and *ost1-3* mutant to foliar ABA spraying (0 μM, 5 μM and 50 μM). Average (± SE, n=9) stomatal conductance before and 56 min after treatment with ABA. Statistically significant differences between post- and pretreatment stomatal conductance values are denoted by * (Repeated measures ANOVA with Tukey post hoc test, $p<0.05$).
