# Supplementary material for: ABA‐mediated regulation of stomatal density is OST1‐independent
Source: Plant Direct. 2018 Sep 19;2(9):e00082. doi: 10.1002/pld3.82 (PMC6508810; doi:10.1002/pld3.82)
Supplement: Supplementary file 2 [file PLD3-2-e00082-s002.pdf]

Dear Plant Direct Editors

Thank you for the comment on the title of our manuscript. As requested we have changed the title of the manuscript to “ABA-mediated regulation of stomatal density is OST1-independent”.

Regards

Mikael

RE: ABA-mediated regulation of stomatal development is OST1-independent

Dear Dr. Brosche:

Thank you for submitting to Plant Direct. We have now finished our evaluation of your revised manuscript. A further small revision is needed before the paper can be accepted for publication in Plant Direct. We ask that you change the title to "ABA-mediated
regulation of stomatal density is OST1-independent", because stomatal development covers many aspects.

When uploading the revised version of this article, please be sure to include the following:

-A tracked changes document with each change highlighted
- A clean version of the latest version of the manuscript

Dear Plant Direct Editors

We are glad to submit to you the revised version of our manuscript 'Complexity of ABA signaling for stomatal development and aperture regulation' by Jalakas et al (please note that we changed the title of the manuscript as indicated below in the detailed comments). We would like to thank the editors and reviewers for their comments and suggested edits, which were helpful in preparing the revised version of our manuscript. We have carefully edited the manuscript according to your comments.

Below is a more detailed response to the specific reviewer/editor comments. Our response is indicated in text with grey background. The manuscript file is provided both as a clean file and a version with all edits highlighted.

With best wishes,

Mikael Brosché

Editor comment:
We have received reviews of manuscript entitled "Complexity of ABA signaling for stomatal development and aperture regulation". Thank you for submitting your work to Plant Direct. It is known that ABA is involved in both stomata development and aperture. The previous results have shown that both mutants in ABA biosynthesis and catabolism affect both stomatal development and response, while OST1 mutation only influences stomatal response to ABA but not stomata development. This work generated double and triple mutants and provided genetic data to support the notion that ABA regulates stomatal density and aperture through OST1-independent and OST1-dependent pathway, respectively. I agree with reviewer two that the experiments were well designed and the data are solid to support your conclusion. I also agree with the first reviewer that your data do not support the conclusion well as summarized in the title of the manuscript. I suggest that you revise the related statements and make the title more informative.

Response:

We agree with reviewer 1 and editor; to make the manuscript title more informative about the key finding we changed the title of our manuscript to "ABA-mediated regulation of stomatal density is OST1-independent"

In addition, minor edits for clarity and to support the main conclusion were introduced into the text on the following places (line numbers refer to the version of the manuscript were the edits are highlighted):

-in the Abstract (lines 28-29).

-in the Introduction (lines 90-92), to more clearly highlight our key finding – that OST1 is not required for stomatal development.

-in the Discussion to more clearly indicate the key role of OST1: lines 211-212; 254, 257-258, 265-266-267.

Reviewer comments:
Reviewer #1:

In previous reports, it has been demonstrated that ABA affects stomatal developments, and OST1 is a key kinase in ABA-induced stomatal closure. In addition, the contribution of stomatal density and apertures in stomatal conductance has been discussed previously (Functional Plant Biology, 2006, 33:1037-1043). Here, Jalakas et al. obtained ost1 aba3 double mutant and ost1 cyp707a1/a3 triple mutant by crossing the aba3 mutant (with lower ABA concentration and higher stomatal density), cyp707a1/a3 mutant (with higher ABA concentration and lower stomatal density), with ost1 mutant. By measuring stomatal conductance, stomatal aperture and density of these mutants, they suggest two signaling pathways in regulating water flux in leaves by ABA: an OST1-independent signaling that regulates stomatal density and an OST1-dependent pathway that regulates rapid changes in stomatal aperture. This work provides insufficient new information for further mechanistic understanding of stomatal development and aperture regulation, and has not provided enough data to support the title "Complexity of ABA signaling for stomatal development and aperture regulation".

Response:

We agree that the previous title of the manuscript did not highlight our key finding, OST1 independent regulation of stomatal development. Hence the title was changed to “ABA-mediated regulation of stomatal density is OST1-independent”.

In addition, as outlined in the response to the Editor, various other minor changes to the text were made to emphasize the key results of the study (related to OST1 function), as well as reducing the emphasis on the complexity of ABA signaling pathways.

Reviewer #2:

The manuscript by Jalakas et al reported the function of ABA concentration and signaling on stomatal development and stomatal aperture regulation. Through comparison of the stomatal conductance, stomatal density and stoamtal aperture among the ost1aba3, ost1-3cyp707a1/a3 and their corresponding single mutant or double mutant plants with altered ABA sensitivity or endogenous ABA concentration, the authors found that ABA concentration regulation of stomatal development is OST1-independent, while ABA regulation of stomatal aperture width is OST1-dependent, mainly through ABA signaling, not by ABA concentration. The manuscript sounds great and provides some novel information for breeding of plants with improved properties in water limiting conditions.

Response:

We thank the reviewer for the kind comments on our manuscript.
